# Impulse Control Disorders and Dopamine-Related Creativity: Pathogenesis and Mechanism, Short Review, and Hypothesis

**DOI:** 10.3389/fneur.2018.01041

**Published:** 2018-12-06

**Authors:** Pedro J. Garcia-Ruiz

**Affiliations:** Movement Disorders Unit, Department of Neurology, Hospital Universitario Fundación Jiménez Díaz, Madrid, Spain

**Keywords:** impulse control disorder, dopamine agonists, genetics, environment, enhanced creativity

## Abstract

Impulse control disorder (ICD), including pathological gambling, hypersexuality, and compulsive shopping has been linked to antiparkinsonian medication, especially dopamine agonists. The mechanism of ICD is not completely clear, but it seems that ICD is the result of an activation of dopamine receptors, mostly D3 in the ventral striatum. Patients treated with dopamine agonists that have preferential affinity for D3 (including ropinirole and pramipexole) are much more prone to develop ICD. In addition, a genetic component is probably present, especially in young patients. Finally, environment and lifestyle may also play a role: those patients engaged in physical, social, and artistic activities are probably less likely to develop ICD compared to those patients with poor physical activity living in isolated environments.

## Introduction

Impulse control disorder (ICD) is currently one of the most frequent and devastating side effects of antiparkinsonian medication. J.A. Molina was the first author to describe gambling as a peculiar and typical manifestation of ICD (1). He found several gamblers among his patients by chance (1); over time, it became clear that ICD was very frequent in Parkinson disease (PD), that this disorder was very complex (2–5); and included several abnormal behaviors such as gambling, hypersexuality, compulsive shopping, kleptomania, and eating disorders (4, 5). It was also clear that ICD was associated with antiparkinsonian drugs, mainly dopamine agonists (6, 7). The relationship of dopamine agonists and ICD has been confirmed in several studies (6–10), most especially in young individuals (11). This review discusses several aspects concerning the pathogenesis and mechanisms of this common and devastating condition.

## Impulse Control Disorder as a Dopaminergic Side Effect

The mechanisms of ICD are not completely clear, but several clues have emerged over time. PD itself does not seem to confer an increased risk for development of ICD (12), thus making ICD mainly a drug-related side effect.

Dopaminergic medication—primarily dopamine agonists (4–11), occasionally MAO-inhibitors (7, 13), and, only rarely, levodopa (14)—has been associated with ICD. Dopamine agonists are clearly related to ICD, not only in PD, but also in restless legs syndrome (10, 15), and occasionally hyperprolactinemia (10, 16).

Although its mechanism is still partially unknown, Castrioto et al. (17) suggested an interesting framework to explain ICD in opposition to apathy in PD. Apathy and ICD (like akinesia and dyskinesia) lie at the opposite ends of a spectrum of dopaminergic tone. Pulsatile dopaminergic medication induces sensitization of the limbic ventral striatum and the motor dorsal striatum. This sensitization may lead to a shift from apathy to ICD (and, from a motor point of view, from bradykinesia to dyskinesia). In this regard, Jimenez-Urbieta et al. suggested that levodopa-related dyskinesias and ICD could be defined as a maladaptation to dopaminergic therapy (18). These elegant and plausible hypotheses certainly explain ICD in the context of PD, but they do not explain the occurrence of ICD in other non-parkinsonian conditions such as restless legs syndrome, in which no dopaminergic neurodegeneration is present. In any case, the contribution of the dopaminergic system to the pathophysiology of ICD is solid (17, 18). In addition, Palermo et al. (19) suggested an interesting neurocognitive approach to ICD; these authors suggest that a fronto-striatal and cingulo-frontal dysfunction may reflect impairment in metacognitive-executive abilities (such as response-inhibition, action monitoring, and error awareness) and promote compulsive repetition of behavior. In this regard ICD could be partly defined as a response-inhibition disability (19).

Dopamine agonists are by far the most frequent drugs associated with ICD (4–11), but there is still an ongoing debate; for some authors, ICD could be defined as a dopamine agonist class effect, with all dopamine agonists sharing this side effect (7). Recently, however, several studies have suggested that some dopamine agonists (including ropinirole and pramipexole) are much more strongly associated with ICD than rotigotine (9, 10) or apomorphine (10). Although the figures vary, in general terms the relative risk of ICD is as follows: pramipexole > ropinirole > rotigotine > apomorphine (9, 10, 20). The reason for this difference is unknown, but according to Seeman (20) those dopamine agonists with preferential affinity for the D3 receptor are much more likely to be associated with ICD compared to other less selective agonists, and in general terms, the relative risk of ICD is proportional to D3 affinity (20). And even so, rotigotine and apomorphine are also associated with ICD (9, 10); in fact, the most severe case of ICD we have ever seen was related to apomorphine, and it seems that there is no dopamine agonist that is entirely free from ICD. Treatment of ICD is a challenge. Reduction and/or suppression of dopamine agonists is usually recommended (18), but ICD is not easily reversible. The substitution of a high affinity dopamine D3 agonist for another less selective dopamine agonists is not always successful. Levy and Lang suggested that previous remote exposure to a dopamine agonist may prime patients to develop ICD with further dopaminergic medication (13). In this regard, dopamine agonists may predispose the striatum to develop ICD and medication-related dyskinesias as well (13, 17, 18). Besides the reduction/withdrawal of dopamine agonists, a plethora of therapeutic measures has been suggested, including atypical neuroleptics such as clozapine and quetiapine (21, 22), anticonvulsants (23), amantadine (24), selective serotonin reuptake inhibitors, and opioid antagonists (25) to mention just a few. There is no solid evidence for the effectiveness of these drugs (25). Recently it has been suggested that intraduodenal infusion of levodopa–carbidopa might help (26), though this measure is probably valid in the presence of an important reduction of dopamine agonist, and in any case, there are also anecdotal reports of ICD after the introduction of levodopa–carbidopa infusion (14). Some other authors suggest that deep brain stimulation (DBS) might be useful for patients with ICD (27); similarly, however, this measure is probably effective only if an important reduction of dopamine agonist is carried out (25, 28). It is important to keep in mind that there are reports of cases of ICD occurring after DBS (25, 28). We have had experience with some parkinsonian patients with ICD submitted for DBS; surgical intervention did not improve their ICD despite a profound reduction of dopamine medication and excellent motor control.

## Genetic Aspects of Impulse Control Disorders

Since not all individuals with PD taking dopamine agonists develop ICD, a genetic component is also likely. In addition, there are similarities between the phenotypic presentation of ICD and that of other reward-based behavioral disorders, including binge-eating disorder, pathological gambling, and substance-use disorder (19, 29).

In the general population, genetic factors might contribute up to 60% of the variance in the risk for substance-use disorders and pathological gambling (30); hence, a genetic component of ICD has been pursued as a viable explanation.

First, although newly diagnosed but still untreated patients with PD do not have an increased risk of developing an ICD when compared to controls (12), certain subpopulations such as younger patients (11) and Parkin mutation carriers (31) do have increased risk.

To date, several polymorphisms of dopaminergic genes have been associated with ICD in PD patients (32–36). However, some findings have challenged this relation, probably due to differences in study design, method of ICD behavior assessment, cohort characteristics, and ethnic background (32–36). The most promising candidate at present is probably the DRD3 single nucleotide variation (SNV) rs6280 (35, 36), which has been associated with ICD in early onset PD in European and Asian patients (35, 36).

In any case, it is evident that multiple factors influence the presence of ICD. Several recent papers found that ICD was mainly associated with an early onset of the disease, dopamine agonist treatment, and the presence of the rs6280 DRD3 SNV (35, 36).

## Impulse Control Disorders, Enhanced Creativity, and Environment

Epidemiological studies revealed that ICD figures vary depending on the country as well as social and economic factors (7–9, 11, 37). Even the characteristics of ICD vary depending on the study (hypersexuality, gambling, compulsive eating, etc., depending on the country) (7–11, 37), hence several environmental factors clearly play a role in the development of the disorder (7, 9, 11, 37).

Another related and interesting aspect is that occasionally, dopamine agonists give rise to enhanced creativity in PD patients, many without previous artistic abilities (38–43); this non-disruptive behavior is described as positive by patients and families. Canesi et al. suggested that artistic-like production might represent the emerging of innate skills in a subset of predisposed patients with PD on dopaminergic therapy (39).

At our center, we have had the chance to follow 10 PD patients with this “newfound talent” and the impact on their lives has been positive, in contrast with the much more frequent ICD. All these patients began their artistic activity after dopaminergic medication (Table 1), most had motor complications including motor fluctuations (7/10), gait freezing (3/10), or dyskinesias (3/10). All but one patient were treated with dopamine agonists including pramipexole (7/9), ropinirole (1/9), or rotigotine (1/9).

**Table 1:** Creativity related to dopaminergic drugs.

**Subject**	**Age/Sex**	**Years**	**Motor Compl**.	**LD**	**Dopamine agonist**	**Artistic activity**
1	69/M	10	F,GF,D	+ (+R)	ROP	Painting, scale models, woodwork
2	70/M	8	GF	+ (+R)	PRM	SCALE models (SHIPS)
3	74/M	10	F,GF	+	PRM	Gardening
4	75/M	8	F	+ (+R)	PRM	Painting
5	67/F	3	–	+ (+R)	PRM	Painting/dance/theater
6	53/F	5	D	+ (+E)	–	Painting
7	71/M	5	F, D	+ (+R)	ROT	Gardening
8	80/M	12	–	+	PRM	Carving, engraving
9	60/M	12	F	+ (+R)	PRM	Scale models (TRAIN)
10	80/F	8	F,GF	+ (+R)	PRM	Painting (>100)

*Motor Comp., Complications; F, Fluctuations; GF, Gait freezing; D, Dyskinesias; LD, Levodopa; ROP, Ropinirole; PRA, Pramipexole; ROT, Rotigotine; R, Rasagiline; E, Entecapone*.

**Figure 1:**
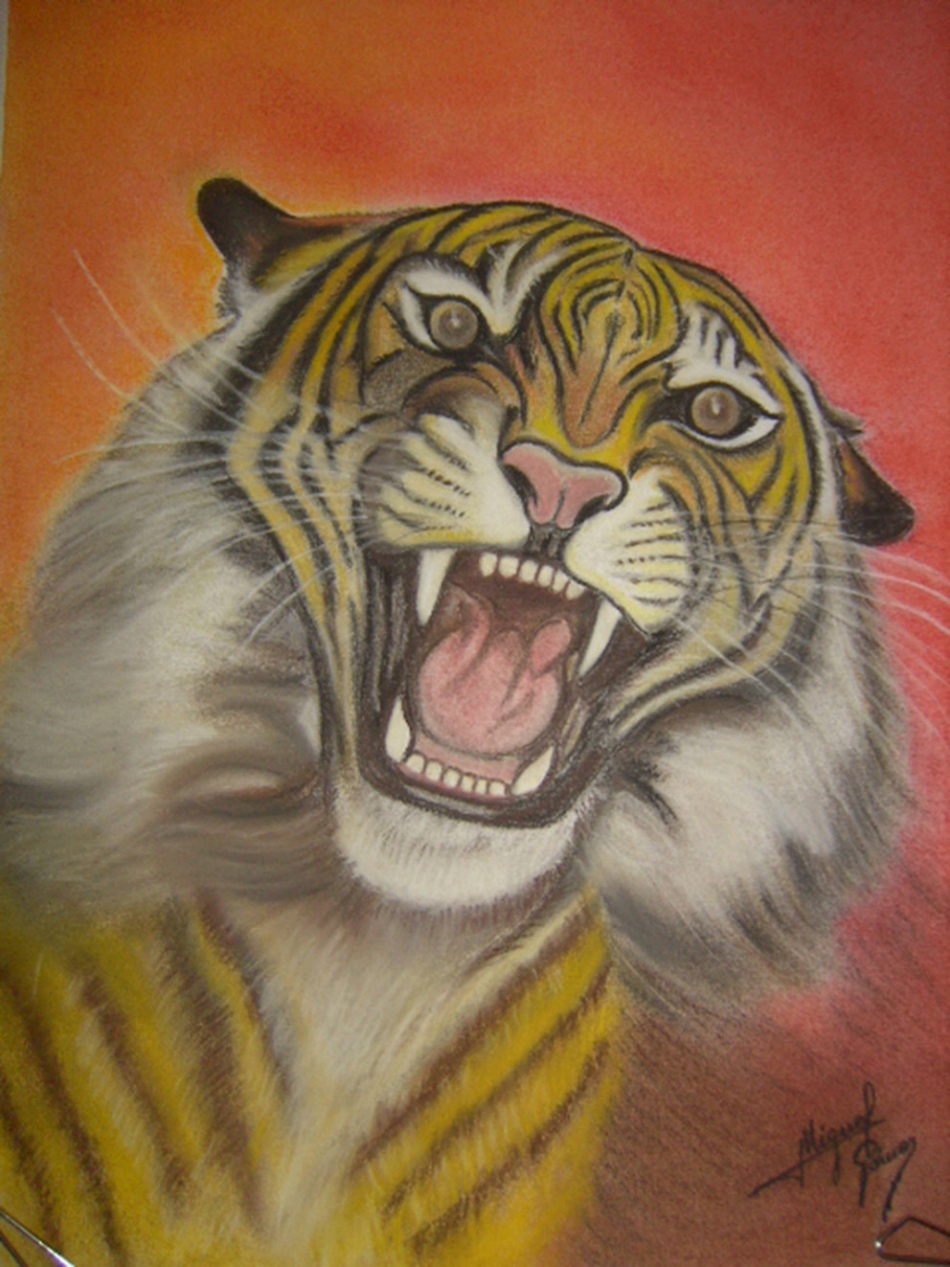
This patient combines realistic portraits and modeling.

**Figure 2:**
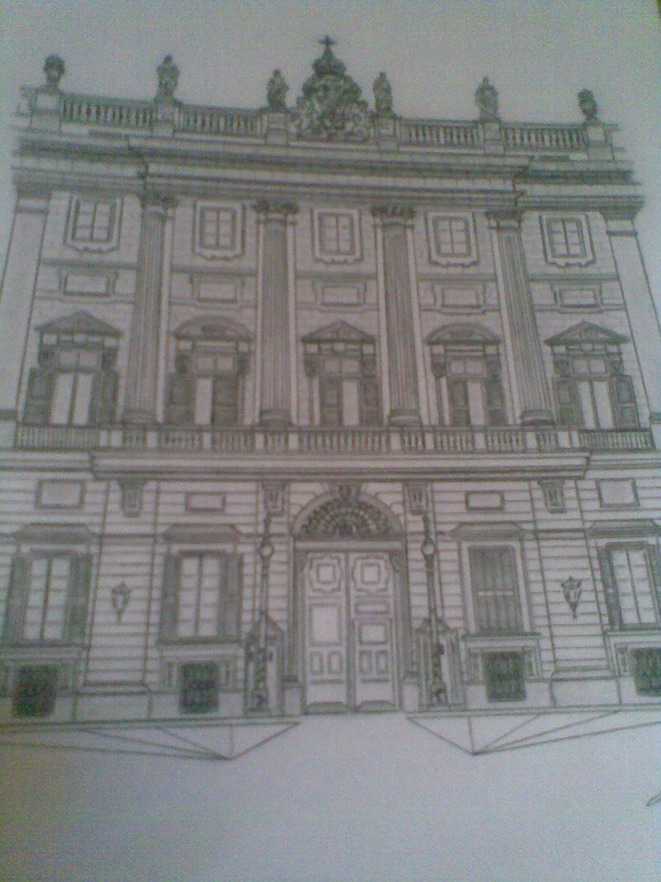
Detailed depiction of a palace (Madrid).

**Figure 3:**
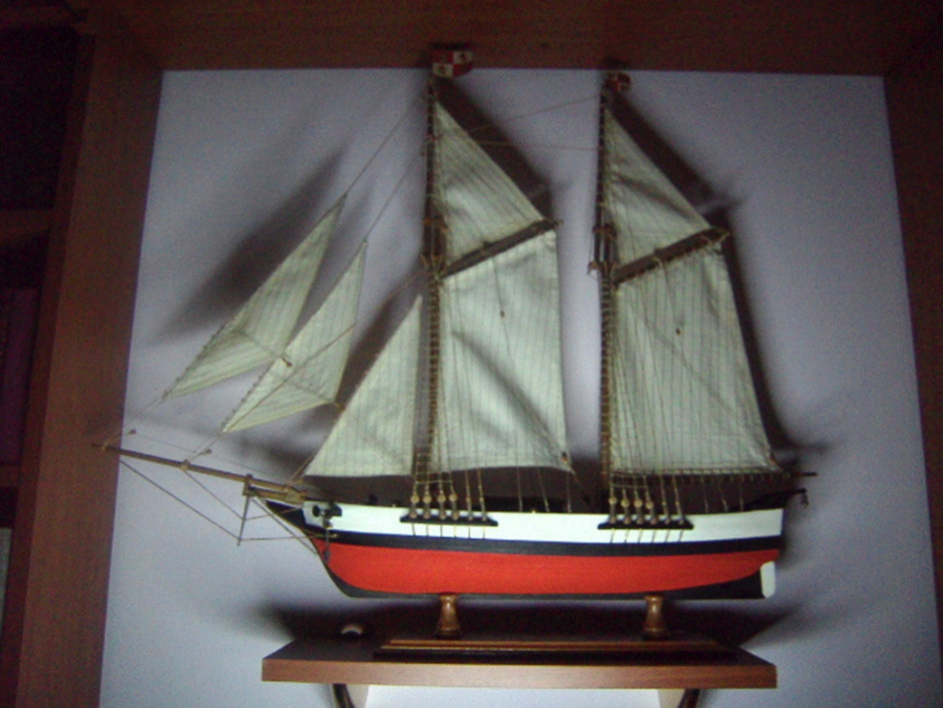
Ship modeling (Schooner), another classic from our patients.

**Figure 4:**
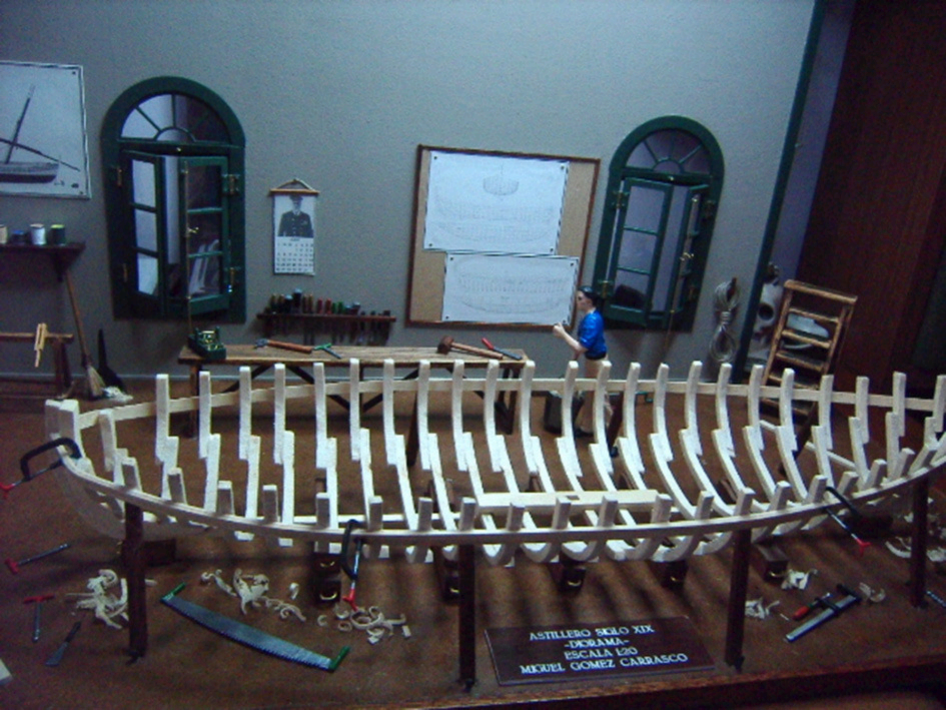
Highly detailed boatyard model.

Most patients with this new artistic activity preferred painting as their main medium, but many were engaged in several activities, usually in combination (Figures 1–4 show some of the art produced by these patients). Some patients began their artistic endeavor after meeting with other subjects already engaged in artistic activities (personal observation). Our hypothesis is that fostering a rich and stimulating environment for patients with PD may contribute to the appearance of this dopamine agonist-related positive phenomenon instead of ICD.

## Conclusion

In summary, ICD is a complex antiparkinsonian medication-related situation most commonly associated with dopamine agonists. Since not all parkinsonian patients suffer from ICD, a genetic component has been pursued. Young patients, including parkin carriers, have increased risk. In addition, environmental factors may also play a role. In any case, early detection of IDC is of paramount importance, as patients must be warned of the onset of this rather frequent side effect.

Recently, a positive, non-disruptive, dopamine agonist-related effect has been noted. Some parkinsonian patients develop enhanced creativity after being treated with dopamine agonists. Facilitating a positive environment (including artistic and cultural activities) for parkinsonian patients may contribute to enhanced creativity instead of ICD. Table 2 summarizes the most relevant points of ICD.

**Table 2:** Impulse control disorders and dopaminergic creativity short review and hypothesis.

Impulse control disorder (ICD) has been linked to antiparkinsonian medication especially dopamine agonists The mechanism of ICD is not completely clear, but activation of dopamine D3 receptor is likely; those patients treated with dopamine agonists with higher affinity to D3 (including ropinirole and pramipexole) are much more prone to develop ICD A genetic component is probably present, especially in young patients The management of ICD includes reduction/suppression of dopamine agonists Occasionally, dopamine agonists enhance creativity and the patients engage in artistic, non-disruptive behavior described as positive by patients and families Probably, the environment influences the apparition of enhanced creativity, our hypothesis is that fostering a rich and stimulating environment for patients with PD may contribute to the appearance of the enhanced creativity phenomenon instead of ICD.

## Author Contributions

PG-R: conception and design, interpretation of data, drafting the submitted material, and critical review.

### Conflict of Interest Statement

PG-R received research support from Allergan and UCB, personal compensation as a consultant/scientific advisory board from Italfarmaco, Britannia, Bial, and Zambon and speaking honoraria from Italfarmaco, UCB, Zambon, Allergan, and Abbvie.
